# Derivation of therapeutic lung spheroid cells from minimally invasive transbronchial pulmonary biopsies

**DOI:** 10.1186/s12931-017-0611-0

**Published:** 2017-06-30

**Authors:** Phuong-Uyen C. Dinh, Jhon Cores, M. Taylor Hensley, Adam C. Vandergriff, Junnan Tang, Tyler A. Allen, Thomas G. Caranasos, Kenneth B. Adler, Leonard J. Lobo, Ke Cheng

**Affiliations:** 1 Department of Molecular Biomedical Sciences and Comparative Medicine Institute, North Carolina State University, 1060 William Moore Drive, RB306, Raleigh, NC 27607 NC USA; 2 Department of Biomedical Engineering, University of North Carolina at Chapel Hill and North Carolina State University, Raleigh/Chapel Hill, NC USA; 3 Department of Cardiology, the First Affiliated Hospital of Zhengzhou University, Zhengzhou, Henan China; 4 Divisions of Cardiothoracic Surgery, University of North Carolina at Chapel Hill, Chapel Hill, NC USA; 5 Pulmonary Diseases and Critical Care Medicine, University of North Carolina at Chapel Hill, Chapel Hill, NC USA

**Keywords:** Pulmonary progenitor cells, Lung spheroid, Stem cell

## Abstract

**Background:**

Resident stem and progenitor cells have been identified in the lung over the last decade, but isolation and culture of these cells remains a challenge. Thus, although these lung stem and progenitor cells provide an ideal source for stem-cell based therapy, mesenchymal stem cells (MSCs) remain the most popular cell therapy product for the treatment of lung diseases. Surgical lung biopsies can be the tissue source but such procedures carry a high risk of mortality.

**Methods:**

In this study we demonstrate that therapeutic lung cells, termed “lung spheroid cells” (LSCs) can be generated from minimally invasive transbronchial lung biopsies using a three-dimensional culture technique. The cells were then characterized by flow cytometry and immunohistochemistry. Angiogenic potential was tested by in-vitro HUVEC tube formation assay. In-vivo bio- distribution of LSCs was examined in athymic nude mice after intravenous delivery.

**Results:**

From one lung biopsy, we are able to derive >50 million LSC cells at Passage 2. These cells were characterized by flow cytometry and immunohistochemistry and were shown to represent a mixture of lung stem cells and supporting cells. When introduced systemically into nude mice, LSCs were retained primarily in the lungs for up to 21 days.

**Conclusion:**

Here, for the first time, we demonstrated that direct culture and expansion of human lung progenitor cells from pulmonary tissues, acquired through a minimally invasive biopsy, is possible and straightforward with a three-dimensional culture technique. These cells could be utilized in long-term expansion of lung progenitor cells and as part of the development of cell-based therapies for the treatment of lung diseases such as chronic obstructive pulmonary disease (COPD) and idiopathic pulmonary fibrosis (IPF).

**Electronic supplementary material:**

The online version of this article (doi:10.1186/s12931-017-0611-0) contains supplementary material, which is available to authorized users.

## Background

The lung is a highly complex organ; it is responsible for respiration but it also acts as a barrier to exterior pathogens and pollutants. It’s composed of over forty different cell types that make up the three major pulmonary regions: tracheobronchial, intralobar airway, and alveolar. The adult lung is a highly quiescent organ; however, after injury or irritation the lung has a remarkable ability to regenerate. Therefore the lung is considered an organ with “facultative” stem/progenitor cell populations [1, 2]. Thanks to lineage tracing, three main stem/progenitor cell populations have been established in the lung. These coordinate the maintenance and regeneration in the three main pulmonary regions [3].

In the proximal trachea, basal cells maintain and give rise to club cells and ciliated cells [4–7]. The club cells found throughout the airway are able to self-renew as well as give rise to ciliated cells. Together the basal and club cells are responsible for maintaining the bronchiolar epithelium [8, 9]. The alveolar epithelium is primarily maintained by alveolar type 2 (AT2) cells, which also have the ability to self-renew and give rise to alveolar type 1 (AT1) cells [10–14]. Under certain conditions club and AT1 cells can de-differentiate back into basal and AT2 cells, respectively [8, 13]. This plasticity makes the lung a good source of therapeutic cells to treat lung disease, but isolation and study of lung stems cells has been extremely difficult, due in large part to the organ’s heterogeneity and complexity.

Cell-based therapy for lung disease has been primarily focused on the use of non-resident stem cells, particularly mesenchymal stromal cells (MSCs), due to their immunoprivileged properties [15–20]. However, MSCs have a very low rate of engraftment in the lungs, as well as a low rate of differentiation into lung cells [21–23], due at least in part to the fact that these cells are extrinsic to the lung. The use of resident lung stem/progenitor cells for cell-based therapy would have a great advantage due to the cells' inherent ability to engraft and survive in a familiar environment. The development of a method(s) to utilize these cells for this purpose would be invaluable. The multicellular spheroid method has been used before to generate cardiac stem cells with therapeutic potential [24, 25]. We have previously demonstrated that regenerative lung spheroid cells (LSCs) could be derived from healthy lung donor tissues, and that these cells have disease-mitigating properties in a mouse model of bleomycin-induced pulmonary fibrosis [26, 27]. However, obtaining lung tissues from patients is not a trivial task. Surgical lung biopsies can provide a large amount of lung tissue, but such procedures are associated with high mortality (3–28%) [28]. In contrast, the transbronchial biopsy procedure is much safer (0.20% mortality) [29], but the amount of tissue recovered from each transbronchial biopsy is very limited, and it is unknown whether lung spheroid cells can be derived from this procedure. Therefore, in this study, we sought to develop a rapid and efficient method to derive therapeutic lung spheroid cells from minimally invasive lung biopsies. We compared LSCs derived from transbronchial biopsies and whole lung tissues for their growth potentials and phenotypes.

## Methods

### Cell Culture

Human LSCs were generated from whole lung (WL) and transbronchial (TB) samples and expanded as described [30]. Briefly, tissue samples were washed with phosphate buffered saline (PBS) (Life Technologies), followed by enzymatic digestion at 37 °C in 5 mg/mL collagenase IV solution (Sigma-Aldrich) for 5 minutes. Iscove’s Modified Dulbecco’s Media (IMDM; Life Technologies) containing 20% fetal bovine serum (FBS; Corning) was then added to the sample to inactivate the collagenase. The tissue samples were further minced into smaller tissue explants (~0.5 × 0.5 mm). Approximately 15–50 pieces of tissue explants were then placed onto a fibronectin-coated plate with approximately 1.5 cm between each explant, and covered with 2 mL of IMDM with 20% FBS overnight. The cultures were maintained in IMDM with 20% FBS and media change was performed every other day. In about seven days, cells started to grow out from the tissue explants. Once these outgrowth cells were about 70–80% confluent, usually around day 17–25, they were harvested after 5–10 minutes of incubation with TryPLE Select™ (Life Technologies). The cells were then seeded into an Ultra-Low attachment flask (Corning) at a density of 100,000 cells/cm^2^ and cultured in IMDM with 10% FBS. Phase-bright lung spheroids started to form in 5–7 days. Lung spheroids were then collected from the low-attachment flasks and re-plated onto fibronectin-coated surfaces to produce adherent LSCs. LSCs were cultured in IMDM with 20% FBS media and passaged every 5–7 days. We used passage 2–5 LSCs for all in-vitro and in-vivo testing. Bronchia/trachea epithelial cell growth medium (Sigma-Aldrich; 511–500) was used for testing effects of media on cell markers.

### Cell population doubling

We started with a known amount of cells plated to a flask. On the next passage, cells are counted and the amount was compared to the original cell count plated. Using these numbers and the known amount of time in between the cell counts, the rate of population doubling can be calculated. This process was repeated for each passage of cells. The following equation was used: $\log \left(\frac{cell\; count\; at\; end\; passage}{cell\; count\; at\; plating}\right)/ \log (2)$.

### Flow cytometry

Cells were washed with MACS flow buffer (MACS, 130-091-222) and permeabilized with BD Cytofix/Cytoperm (BD, 554714) prior to incubation with antibodies. Cells were labeled for antibodies against CD90 (Abcam, ab3105; Abcam, ab124527; Abcam, ab23894; BD, 555595), CD105 (Abcam, ab107595; Abcam, ab2529; Abcam, ab11414; R&D Systems, Fab10971p), Pro-SPC (Bioss, bs 10067R; Abcam, ab40879), CCSP (Abcam, ab171957), Epcam (Abcam, ab71916, Abcam, ab168828; Life Technologies, a15755), and Aqp5 (Abcam, ab78486; Abcam, ab85905) and detected by Alexa Fluor 488 (Abcam, ab150117, ab150077) or fluorescein isothiocyanate (FitC) (Abcam, ab6840) secondary antibodies. Both unstained and isotype controls (Abcam, ab18419; BD, 559320; Abcam, ab125938) were utilized as controls. Human adipose-derived mesenchymal stem cells (AD-MSCs) were obtained from Lonza. Flow Cytometry was performed on the CytoFlex (Beckman Coulter, Indianapolis, IN) and analyzed using FCS Express (De Novo Software, Glendale, CA) or CytExpert ((Beckman Coulter, Indianapolis, IN).

### Immunocytochemistry

Cells were plated onto fibronectin-coated 4-well chamber slides (Millipore; PEZGS0416). Once the desired confluency was achieved the slides were fixed in 4% paraformaldehyde (PFA) (Electron Microscopy Sciences; 15710) followed by permeabilization and blocking with Dako Protein blocking solution (DAKO; X0909) containing 0.1% saponin (Sigma-Aldrich; 47036) prior to immunocytochemistry. Cells were stained for antibodies against CD90, CD105, Pro-SPC, CCSP, Epcam, and Aqp5 and detected by Alexa Fluor 488 or FitC secondary antibodies. Slides were imaged on a fluorescent microscope (Olympus; Olympus IX81, Center Valley PA).

### Generation of heat-map images for immunostaining

Single channel immunostained images of the spheroids were imported into ImageJ. The Rainbow RGB lookup table was applied to each image to visualize cell marker density distribution throughout the spheroids. A plot profile histogram was then generated using the rainbow RGB image.

### Tube formation study

Human umbilical vein endothelial cells (HUVECs; American Type Culture Collection) were seeded onto Matrigel in a 96-well plate at a density of 2 × 10^4^ cells per well with 100 μl of plain IMDM, LSC conditioned media, or adipose derived-MSC conditioned media. After 5 h, the wells were imaged with the Nikon TE-200 (Nikon, Tokyo, Japan). The average tube length was measured with NIH ImageJ software.

### Biodistribution of LSCs after intravenous infusion

All studies were in compliance with the requirements from the Institutional Animal Care and Use Committee at North Carolina State University. Athymic nude mice (*n* = 10; Crl:NU(NCr)-Foxn1^nu^; Charles River Laboratories) were intravenously injected with 5 × 10^6^ LSCs from passage 1–3 suspended in 300 μL of a 1:10 heparin/PBS solution. Two different LSC lines were used and mice were randomly assigned to the two different lines. Before injection, all cells were labeled with the lipophilic tracer DiD (Invitrogen) as per their protocol for subsequent live imaging using the Xenogen Live Imager. After taking baseline images, mice were injected with the DiD-labeled LSCs. Subsequent images were taken at 1, 4, 7, 11, and 20-day time points, after which the mice were euthanized. The hearts, lungs, livers, spleens, and kidneys were removed and imaged separately to allow examination without interference from other tissues.

For each nude mouse, an image showing luminescence was analyzed using the region of interest (ROI) tools from the tools palette window of the Living Image 4.2 software package. First, the image was adjusted so that each subject’s radiant efficiency reading was set to a manually fixed scale (a minimum and maximum range). This is important for visual comparisons of the different time points but not necessary for using ROI tools. The color table was set to Rainbow 2 and reversed for visual clarity of the signal. A circular ROI was chosen and the diameter was fixed to encompass the entire signal range. The same diameter was used for every nude mouse image. One ROI was placed around the lungs and another around the liver of each subject. As the signal began to fade from the organs, a heuristic technique was used to separate the liver from the lungs. The liver signal remained quite discernable so it was used as a starting point. The upper extremity of the liver signal was used as the outer-most edge of the lower-most region of the lung signal. The two ROI perimeters were set so that they never touched. The measurement type was set to radiant efficiency and was then measured. The average radiant efficiency output was saved and exported to Excel where it could be compared across the time points.

### Statistical analysis

All results are expressed as mean ± standard deviation (SD) and Gaussian distribution of data is tested using Kolmogorov-Smirnov test and/or D’Agostino and Pearson omnibus normality test. Comparison between two groups was conducted by two-tailed Student’s *t*-test. One-way ANOVA was used for comparison among three or more groups with Bonferroni post hoc correction. Differences were considered statistically significant at *P*-values <0.05.

## Results

### Lung spheroid cells can be expanded from minimally invasive transbronchial biopsies

**Fig. 1:**
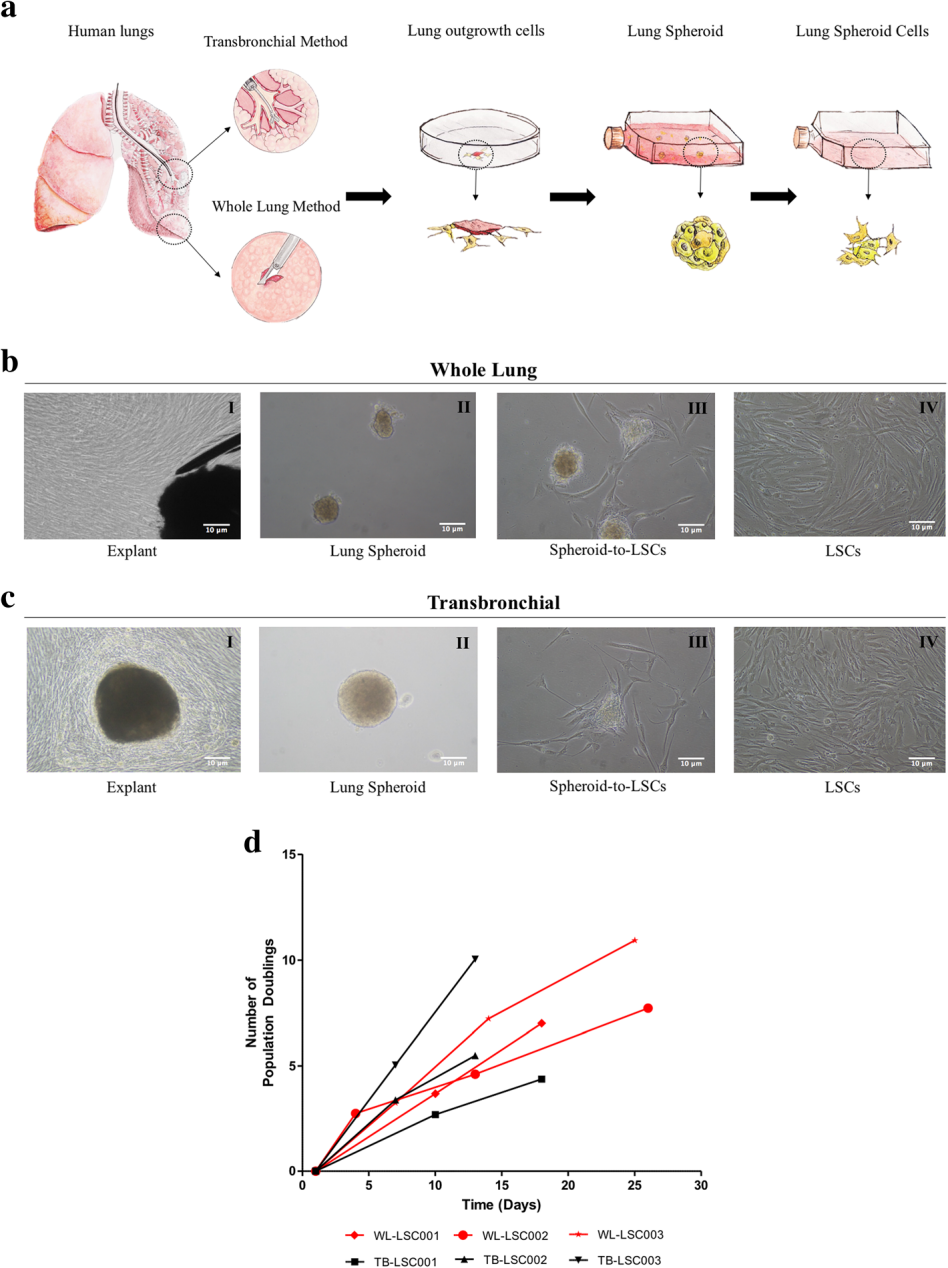
Growth potential lung spheroid cells derived from whole lung and biopsy specimens. **a**: Schematic showing the protocol to derive lung spheroid cells (LSCs). **b** I-IV: Bright field image of each stage of WL-LSC generation. **b**(I): Explant tissue in the middle surrounded by outgrowth of cells. **b**(II): Lung spheroids formed from explant-derived cells (EDCs). **b**(III): Plated spheres onto fibronectin coated surface allowing lung spheroid cells to grow out from the spheroids. **b**(IV): Expansion of LSCs in adherent culture. **c** I-IV: Bright field image of each stage of TB-LSC generation. **d**: Cumulative population doubling for TB-LSCs and WL-LSCs from three different donors. Scale Bar = 10 μm

Whole lung (WL) tissue samples and transbronchial (TB) biopsy samples were obtained from the Cystic Fibrosis Center and Pulmonary Diseases Research and Treatment Center at the University of North Carolina at Chapel Hill. Donor comorbidity information is presented in Table 1. Samples were taken from the distal lung region of either whole donor lungs or transbronchial biopsies. Tissue samples were processed using the three-dimensional suspension culture method (Fig. 1a). Both sample types showed an outgrowth of phase-bright and stromal-like cells approximately seven days post-plating (Fig. 1b-i). Those outgrowth cells (or explant-derived cells, EDCs) were then seeded onto ultra-low attachment flasks; under these culture conditions the outgrowth cells spontaneously form a three-dimensional cell suspension termed a “lung spheroid” (Fig. 1b-II). When plated onto fibronectin-coated surfaces, these lung spheroids generated 10–20 million cells that we termed “lung spheroid cells” or LSCs (Fig. 1b-III-IV).

**Table 1:** Donor Table

	Sex	Age	Race	Smoker	Cause of Death
WL Donor 1	Female	50	Hispanic	No	Anoxia 2^nd^ Cardiovascular
WL Donor 2	Female	52	Black	No	Cerebrovascular Accident
WL Donor 3	Male	18	Hispanic	No	Head Trauma 2^nd^ Self-Inflicted Gunshot Wound
	Sex	Age	Race	Smoker	Additional Info
TB Donor 1	Male	46	Hispanic	No	Fibrosing NSIP; Received Lung Transplant
TB Donor 2	Male	76	White	No	Mixed connective tissue associated NSIP
TB Donor 3	Male	64	White	Yes	IPF; Received Lung Transplant

Compared to WL tissue samples, TB samples required more time for the outgrowth of cells from the explant to reach the desired confluency to process into spheres. However, once the cells reached the sphere phase, the LSCs produced from both sample types showed similar growth rates, with 5–10 population doublings every seven to fifteen days (Fig. 1c-d). This growth rate was maintained for up to 7 passages, as long as 30 days with appropriate freeze-thawing from cryopreservation and long-term cell culture. From one TB lung biopsy, we were able to derive >50 million LSCs at Passage 2. This sustainable growth from TB samples indicates that therapeutic lung cells may be derived and expanded from a small sized tissue biopsy sample.

### Lung spheroids contain heterogeneous cell populations

**Fig. 2:**
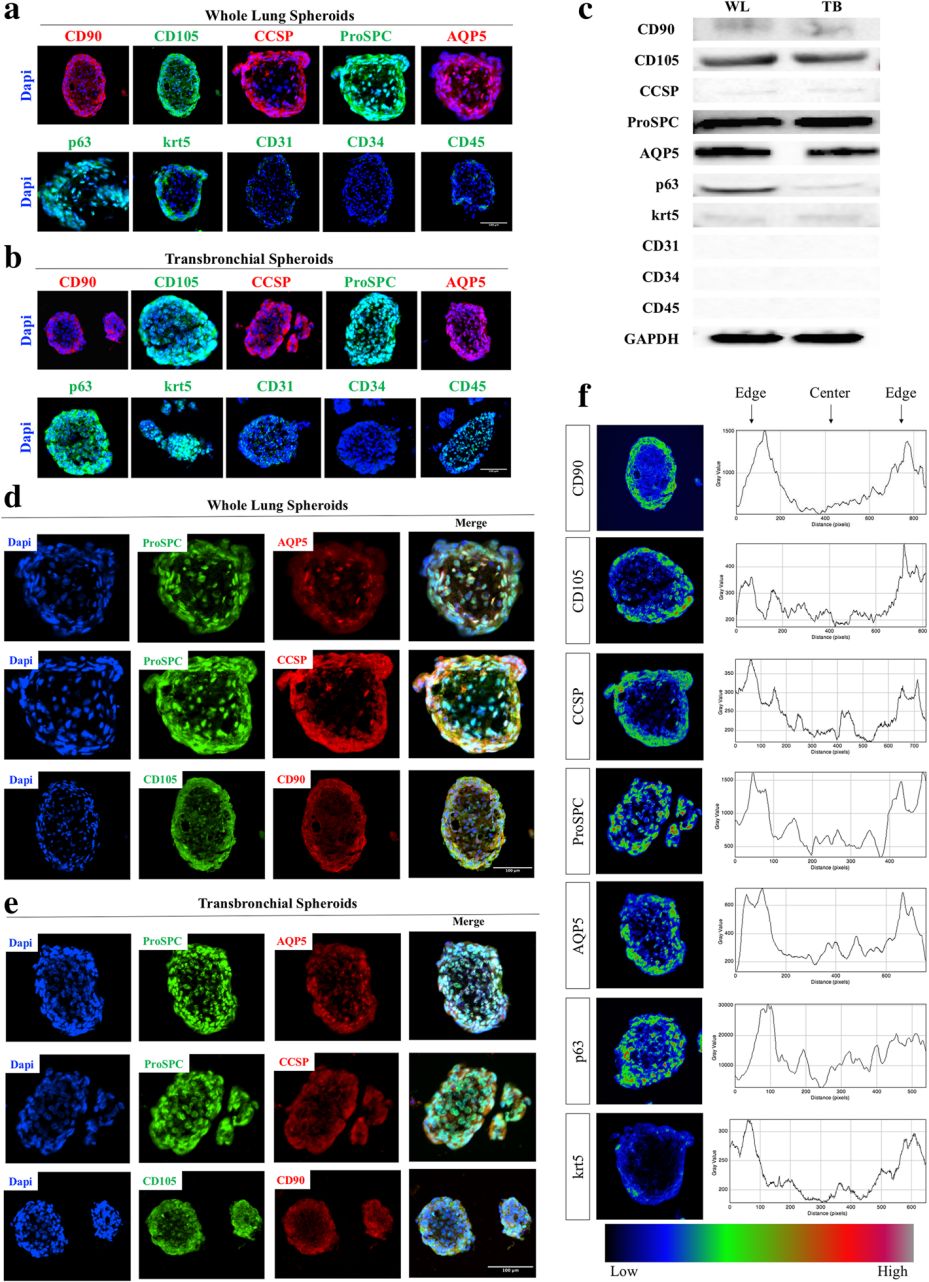
Phenotype analysis of lung spheroids. **a**-**b**: Representative immunohistochemistry staining of WL-Spheroids (**a**) and TB-Spheroids (**b**) for expression of CD90 in red, CD105 in green, CCSP in red, ProSPC in green, AQP5 in red, p63 in green, krt5 in green, CD31 in green, CD34 in green, CD45 and Dapi in blue. C: Western blot of WL and TB-Spheroids. **d**-**e**: Double staining of WL-Spheroids (**d**) and TB-Spheroids (**e**) for co-expression of ProSPC/AQP5, ProSPC/CCSP and CD90/CD105. **f**: Expression intensity heat maps and corresponding histograms visualizing the distribution of the markers within the spheroids. Immunostaining negative controls are provided in Additional file 3: Figure S3. For all spheroids imaged *n* ≥ 3. Scale Bar = 100 μm

Immunocytochemistry displays positive CD90, CD105, CCSP, ProSPC, AQP5, p63 and krt5 cell markers and negative CD31, CD34 and CD45 markers in both whole lung and transbronchial spheroids (Fig. 2a-b). This is supported by western blot of the same markers (Fig. 2c). Double staining revealed double positive populations of ProSPC and AQP5, ProSPC and CCSP, and CD105 and CD90 (Fig. 2d-e). Heat maps were generated from each positive marker to examine the spherical distribution of the markers showing the supporting cell types such as CD90, CD105, and krt5 primarily at the sphere edge and CCSP, ProSPC, AQP5 and p63 throughout the sphere (Fig. 2f).

### Lung spheroid cells exhibit a complex heterogeneous cell phenotype

**Fig. 3:**
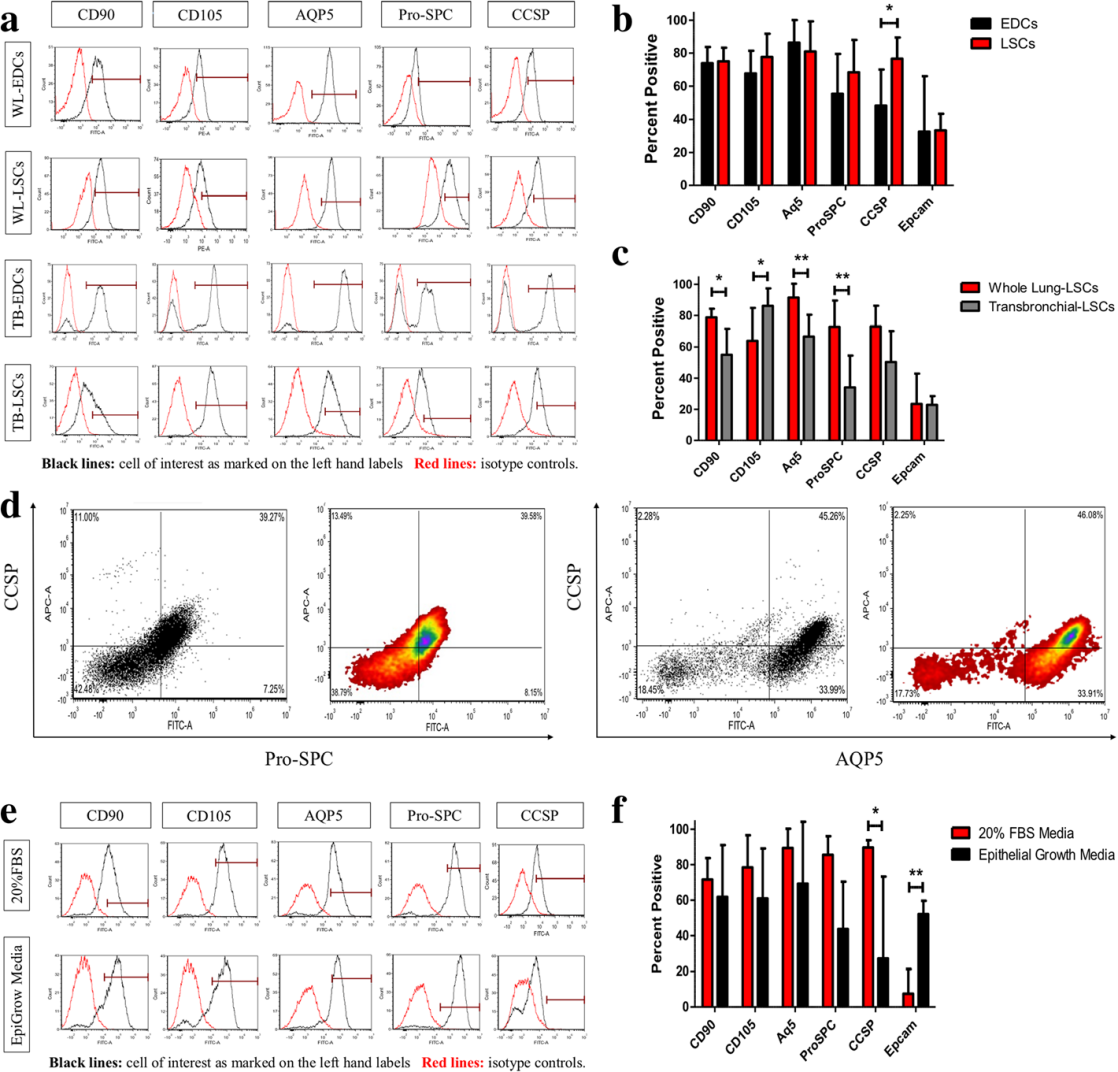
Phenotype Analysis of lung spheroid cells. **a**: Representative flow cytometry histograms of WL-EDCs, WL-LSCs, and TB-LSCs for expression of CD90, CD105, AQP5, Pro-SPC and CCSP. Black lines in the histograms are the cell of interest as marked on the left hand labels. Red lines in the histograms are the unstained or isotype controls. **b**-**c**: Cumulative data for the expression CD90, CD105, AQP5, Pro-SPC, CCSP and Epcam showing the expression change between **b**) WL-EDCs to WL-LSCs (*n* = 5–12) and **c**) WL-LSCs and TB-LSCs (*n* = 7-12). **d**: Double stained flow cytometry plot of CCSP verse ProSPC and CCSP verse AQP5. **e**: Representative flow cytometry histograms of WL-LSCs in either IMDM with 20% FBS or epithelial cell growth medium for expression of CD90, CD105, A15, Pro-SPC, and CCSP. Black lines in the histograms are the cell of interest as marked on the left hand labels. Red lines in the histograms are the unstained or isotype controls. **f**: Cumulative data for the expression CD90, CD105, A15, Pro-SPC, CCSP, and Epcam (*n* = 3-4). Data represented as mean ± SD. * *p* < 0.05; **p < 0.01

Flow cytometry revealed several positive populations of lung progenitor cells (e.g. Club Cells [CCSP] and AT2 cells [Pro-SPC]) along with stromal-like supporting cells (e.g. CD90, CD105) (Fig. 3a). WL-LSCs flow analysis showed the cells were 73.1 ± 5.4% CCSP^+^, 92.3 ± 4.1% AQP5^+^, 72.8 ± 5.3% Pro-SPC^+^, 78.8 ± 1.8% CD90^+^, 63.9 ± 6.1% CD105^+^, and 23.5 ± 6.9% Epcam^+^ (Fig. 3b). There was no significant change in the supporting cell populations from EDCs to the final LSCs, but there was a clear increase in the progenitor cell populations from the EDC stage to LSCs, suggesting three-dimensional culture of lung spheroids may have increased the “stemness” of the cells. There was approximately a 37.2% increase in the proportion of AT2 cells and 36.1% increase in the proportion of club cells from the EDC to LSC stage. There was also a slight but non-significant decrease in the proportion of AT1 cells.

TB-LSCs are also positive for the same markers as WL-LSCs, however, there is a difference in the percentage of positive cell populations (Fig. 3a; c). The levels of CD90^+^ cells decreased by 23.8%, but CD105^+^ cells increased by 22.3%. Lung secretory (CCSP) and alveolar (Pro-SPC and AQP5) markers decreased 22.7%, 38.8%, and 25.7% respectively. Overall, the phenotype of TB-derived LSCs was slightly different from WL-derived LSCs. Double stained flow cytometry reveled a double positive population of lung secretory (CCSP) and alveolar (Pro-SPC and AQP5) markers at approximately 39% and 45% respectively (Fig. 3d). Further analysis of double labeled cells for mesenchymal (CD105) and epithelial (ProSPC) markers showed a double positive population of approximately 38.29% (Additional file 1: Figure S1).

LSC phenotype was further supported by immunocytochemistry which showed the cells are positive for CCSP^+^, AQP5^+^, ProSPC^+^, CD90^+^, CD105^+^, and Epcam^+^ (data not shown). Taken together, these findings show that the LSCs contain a wide mixture of cells including club cells, AT1 cells, and AT2 cells, as well as CD90^+^ and CD105^+^ stromal-origin cells. It has also been previously reported that both cardiac and lung spheroids exhibit progenitor cells in the core of the spheres, surrounded by supporting cells such as CD90^+^ and CD105^+^ cells. This arrangement of cells resembles a stem cell niche environment, necessary to regulate stem cell behavior [30].

When LSCs were cultured with bronchia/trachea epithelial cell growth medium (Sigma-Aldrich) there was a three-fold increase in Epcam expression (Fig. 3e-f) suggesting differentiation of progenitor cells into mature lung cells.

### Lung spheroid cells promote endothelial cell tube formation in-vitro

**Figure 4:**
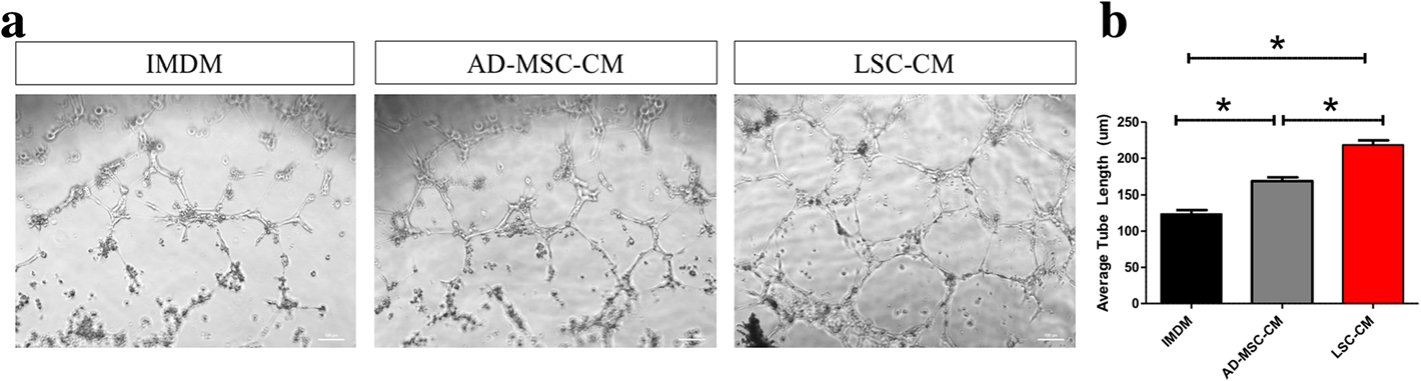
Lung spheroid cells promote endothelial cell tube formation in-vitro. **a**: Bright field image of HUVEC cells in IMDM, adipose-derived mesenchymal stromal cell conditioned media (AD-MSC-CM) and lung spheroid cell conditioned media (LSC-CM). **b**: Cumulative data of average tube lengths (μm). Data represented as mean ± SEM. * *p* < 0.05 Scale Bar = 100 μm

A HUVEC tube formation assay was performed to determine the angiogenic potential of the two LSC types compared to human adipose-derived MSCs (AD-MSCs) (Fig. 4a). Human endothelial cells were seeded on Matrigel (BD Biosciences) and cultured with control medium (IMDM), LSC conditioned medium, or human AD-MSC-conditioned medium. The tube formations of human endothelial cells on Matrigel suggested pro-angiogenic properties in LSC-conditioned medium (Fig. 4b).

### Biodistribution of intravenously-injected lung spheroid cells

**Fig. 5:**
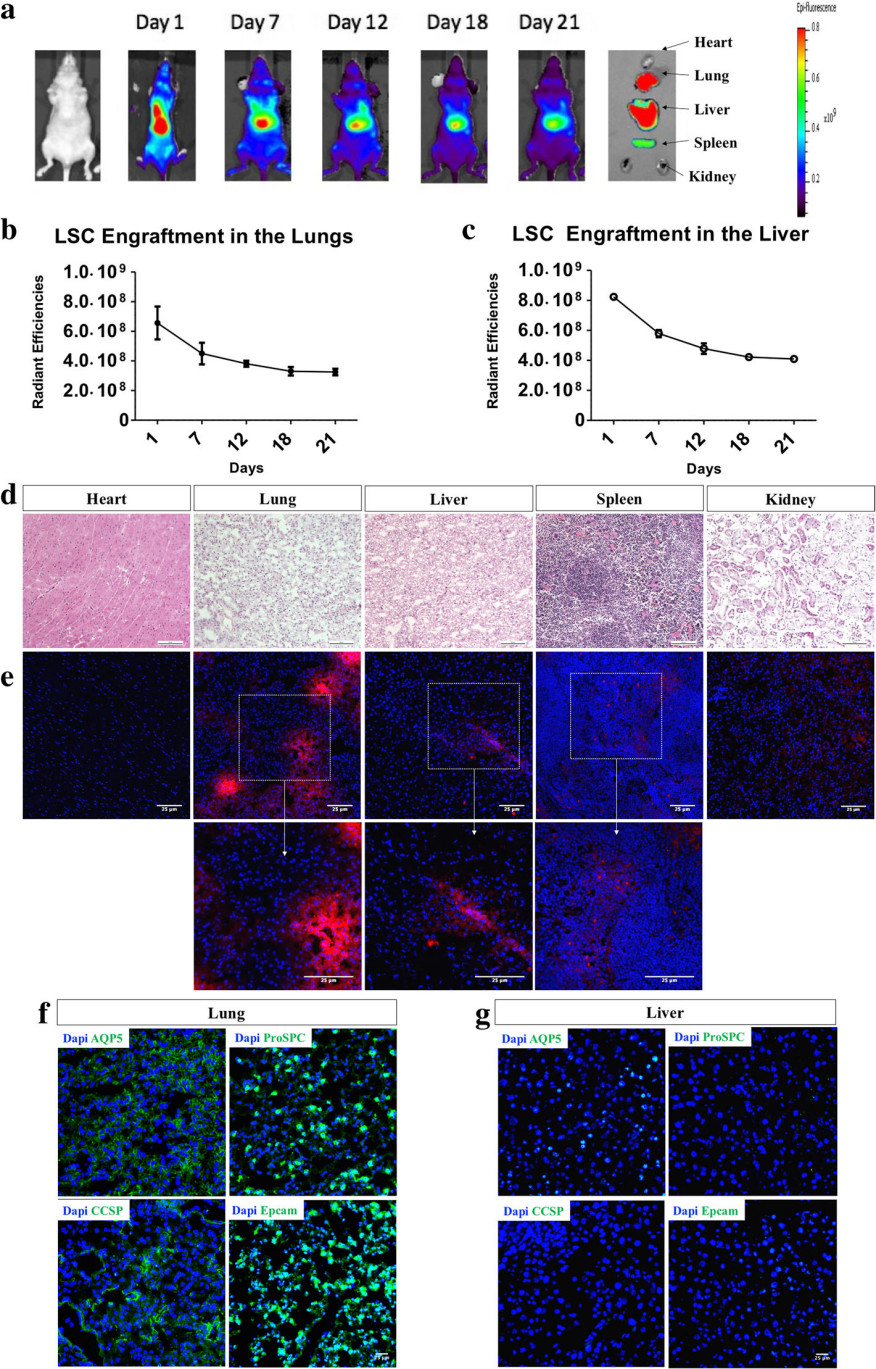
Biodistribution of LSCs in-vivo after intravenous injection. **a**: Representative in-vivo imaging of mice intravenously injected with LSCs at day 1, 7, 12, 18 and 21 along with final organ harvest of heart, lungs, liver, spleen and kidneys. **b**-**c**: Cumulative data for radiant efficiencies in the lungs sand liver. **d**-**e**: Representative hematoxylin and eosin staining (**d**) and immunohistochemistry (**e**) of heart, lung, liver, spleen and kidneys. Data represented as mean ± SD. **f**-**g**: Immunostaining of lung and liver tissue for lung specific markers AQP5, ProSPC, CCSP and Epcam. **d**: Scale Bar = 100 μm. **e**-**g**: Scale Bar = 25 μm. Immunostaining negative controls are provided in Additional file 4: Figure S4.

Before clinical studies of LSCs are conducted in humans, a major question to address related to where the cells seed after systemic delivery. To determine where the cells will target and how long they can be retained once administered in-vivo, we fluorescently labeled LSCs with DiD and administered 5 × 10^6^ cells via the tail vain of athymic nude mice. The mice were imaged at 24 h., 7 days, 12 days, 18 days, and 21 days’ post injection (Fig. 5a). After the final day 21, an image was acquired, and the mice were then sacrificed and organs harvested. At Day 1 post injection, most of the cell signal was found in the lungs and liver, (Fig. 5b-c), and this persisted up to the endpoint at day 21. With such a large dose of infusion (5 million cells in a mouse can be extrapolated to nearly 5 billion cells for a human), no tumor formation was detected in any of the mice that received LSCs. Hematoxylin & Eosin staining further confirmed the absence of tumors in all harvested organs (Fig. 5d). Immunohistochemistry confirms the presences of engrafted LSCs labeled with DiD in the lungs with DiD labeled cell debris and/or secreted factors in the liver and spleen (Fig. 5e). Lung and liver tissues were further examined for LSC phenotypes, showing positive lung alveolar (AQP5 and ProSPC), secretory (CCSP) and epithelial (Epcam) markers in the lung tissue (Fig. 5f). However, these same markers are absent in liver tissues (Fig. 5g). It is worth noting that despite the use of two different cell lines were used for the intravenous injection, of cells no differences were found in the overall cell fate or the rate at which the signal strength subsided with time. Altogether, these datasets indicated that intravenous delivery is an effective method to deliver LSCs to the lungs.

## Discussion

Lung disease remains one of the top causes of morbidity and mortality worldwide [31]. Chronic and degenerative diseases of the airway and alveolar tissues, such as chronic obstructive pulmonary disease (COPD) and idiopathic pulmonary fibrosis (IPF), are particularly devastating and to date have no cure [32, 33]. Despite advances in supportive care and symptomatic treatments, allogeneic lung transplantation is the only effective treatment for these disorders, but the procedure is highly complicated and highly invasive. Complicating this fact is the lifelong immunosuppression required to prevent rejection. Further, lung transplantation has a high five-year mortality rate at approximately 50% [34]. Therefore, new treatment paradigms are desperately needed.

Stem cell-based therapy appears to be a potential major advancement in treating these lung diseases. Major progress has been made in the field of lung regenerative medicine in the past few decades as various stem and progenitor cell populations have been identified and characterized, such as bronchioalveolar stem cells (BASC) [35], alveolar bipotential progenitor (BP) cells [3], and other putative distal lung stem and progenitor cell populations. This provides cells for potential new approaches for treating different lung diseases. Although these stem and progenitor cell populations have been defined, it remains difficult to isolate pure populations of these cells due to a lack of specificity in discreaning surface markers, since many markers are shared between these cell populations. Therefore, mesenchymal stromal cells remain the preferred choice for cell-based therapy in several lung diseases due to their ease of isolation and production, despite the identification of resident lung stem and progenitor cells.

It is important to keep in mind that stem cells do not act alone, but rather interact with surrounding cells (i.e. niche) to perform their necessary functions in both homeostasis and in response to injury. Multicellular spheroids have been used for neural and cardiac stem cells [30, 36, 37] with great success. We have recently shown that lung spheroid cells have regenerative abilities in treating early stage pulmonary fibrosis in a murine model [26]. In the present study, we present two distinct sources for LSC generations using a simple and robust method. Whether generated from whole lung donors or a small transbronchial biopsy, we are able to derive therapeutic cells from both tissue sources. However, it should be noted that whole lung tissue sources were from deceased donors with no history of lung disease (i.e. healthy lungs) while transbronchial tissue sources were from donors suffering advance lung disease (Table 1). This fundamental difference in the tissue source could account for the significant phenotypic difference in the proportion of mesenchymal (CD90 and CD105) and alveolar (AQP5 and ProSPC) markers (Fig. 3 a & c); however, regardless of tissue source, both WL and TB-LSCs express the same overall phenotypes and growth potential (Figs. 1–3). The cell yield and growth potential were comparable for both types of LSCs and are suitable for clinical applications (Fig. 1). The ease of our method is due in large part to the lack of segregation of different cell types, precluding the need for cell sorting. In addition, our method of lung spheroid generation is without bias for any particular cell type (Figs. 2; 3a-c). Therefore, LSCs express a heterogeneous phenotype of both mesenchymal and epithelial markers. We believe the stem and progenitor cells are important for regeneration, and their effect is maximized when non-stem epithelial and mesenchymal cells provide the necessary niche environment for proper stem cell function (Fig. 2). This is especially true for the stromal-like supporting cells, as it’s been shown that fibroblasts and growth factors, such as FGF 9 and 10 can regulate stem cell homeostasis and activation through Wnt signaling [38–41].

The lung is characterized as having “facultative” progenitor cell populations where differentiated cells such as club cells may be induced to re-enter the cell cycle and proliferate in response to stimuli [42]. This type of response is similar to the liver which has the ability to regenerate in response to injury, but is otherwise quiescent. This is different from organs with high cellular turnover that require a dedicated stem cell population, such as the intestine or skin and hair; or organs like the brain which have limited ability to regenerate even after injury. As shown via flow cytometry, the markers for lung secretory (CCSP) and alveolar (Pro-SPC) cells, widely accepted as “facultative” lung progenitor cells, showed a significant increase from explants (EDC) to LSCs (Fig. 3b). This suggests that progenitor cell populations are enriched through the 3-dimensional spheroid culture, perhaps by recapitulating the natural niche environment of the cells. The high percentage of both club and AT2 cells suggest that there is a subpopulation that expresses both markers, which is a hallmark of BASC. There also appears to be an overlap in AT1 and AT2 cell expression, which could be due to the presence of BP cells or intermediate cells in AT2 to AT1 differentiation. It’s also been suggested that AT1 cells have phenotypic plasticity and may not be terminal cells as commonly believed [14]. AT1 cells cultured on fibronectin surfaces in 20% FBS without additional growth factors, similar to the culture condition used in this study, have been shown to proliferate and express phenotypic markers of other cell types such as AT2 cells (Fig. 3). AT1 cells cultured in keratinocyte growth factor have also been shown to lose expression of AT1 markers while reacquiring AT2 markers.

As an important in vitro potency indicator of cell therapy, we performed an endothelial tube formation assay to demonstrate the therapeutic potential of WL-LSCs and TB-LSCs in comparison to MSCs (Fig. 4). We showed that LSCs outperformed AD-MSCs in their angiogenic ability, as shown through endothelial cell tube formation. In vivo, we were able to show, using fluorescently labeled LSCs, that the majority of the cells could be seen in the lungs, where they persisted for up to 21 days (Fig. 5a-g). Even though the liver has high fluorescence, immunostaining shows the absence of cells engrafted in the liver as compared to cell clusters found in the lung (Fig. 5e). This suggests that the high fluorescence in the liver may be due to the natural auto-fluorescent nature of the liver, shown in Additional file 2: Figure S2, and/or a combination of a leak in the fluorescence tracer and cell debris from dead cells that were labeled with the fluorescence tracer that migrated and were subsequently taken up by the liver. Further immunostaining for lung specific markers shows that if any LSCs were to engraft in the liver, they do not retain lung phenotype (Fig. 5f-g). There was also a large amount of off-target cell migration to the liver, but otherwise no complications or tumor formation were observed in any animal subjects throughout the study.

## Conclusions

The results show that lung cells have great plasticity, due in large part to cell culture conditions and crosstalk between cells and between cells and their environment. Thus, lung stem and progenitor cell “organoids/spheres,” with their various niche cells, can be considered superior to pure stem cell populations because they provide the proper cellular support and allow cell signaling. There remains much to be elucidated about the dynamic feedback between stem cells and their niche and how feasible and effective these cells are at treating severely damaged lung epithelium. Lung spheroids and lung spheroid cells provide a new avenue to explore those questions.

Thus, to the best of our knowledge, we are the first to derive lung spheroid cells containing potential therapeutic lung cells from minimally invasive transbronchial biopsy specimens. Through our simple and highly reproducible three-dimensional culture method, therapeutic lung cells can be generated from small biopsy sized tissues in high efficiency and in clinically relevant numbers. Future studies will focus on the therapeutic potential of transbronchial biopsy-derived lung spheroid cells in animal models of lung diseases.

## Additional files

Additional file 1: Figure S1. Double stained LSCs shows mixed phenotype of mesenchymal and epithelial markers.

Additional file 2: Figure S2. Representative in-vivo imaging of control athymic nude mice showing auto-fluorescence.

Additional file 3: Figure S3. Negative controls of all immunostaining for phenotype analysis of lung spheroids.

Additional file 4: Figure S4. Negative controls of all immunostaining for biodistribution of LSCs in-vivo after intravenous injection.

## Abbreviations

AQP5: Aquaporin 5
AT1: Alveolar type 1
AT2: Alveolar type 2
BASC: Bronchioalveolar stem cells
BP: Bipotential progenitor
CCSP: Club cell secretory protein
COPD: Chronic obstructive pulmonary disease
EDC: Explant derived cells
IMDM: Iscove’s Modified Dulbecco’s Medium
IPF: Idiopathic pulmonary fibrosis
LSC: Lung spheroid cells
MSC: Mesenchymal stem cells
Pro-SPC: Pro-Surfactant Protein C
TB: Transbronchial
WL: Whole lung
