# Supplementary figures and images for: Derivation of therapeutic lung spheroid cells from minimally invasive transbronchial pulmonary biopsies
Source: Respir Res. 2017 Jun 30;18:132. doi: 10.1186/s12931-017-0611-0 (PMC5493087; doi:10.1186/s12931-017-0611-0)

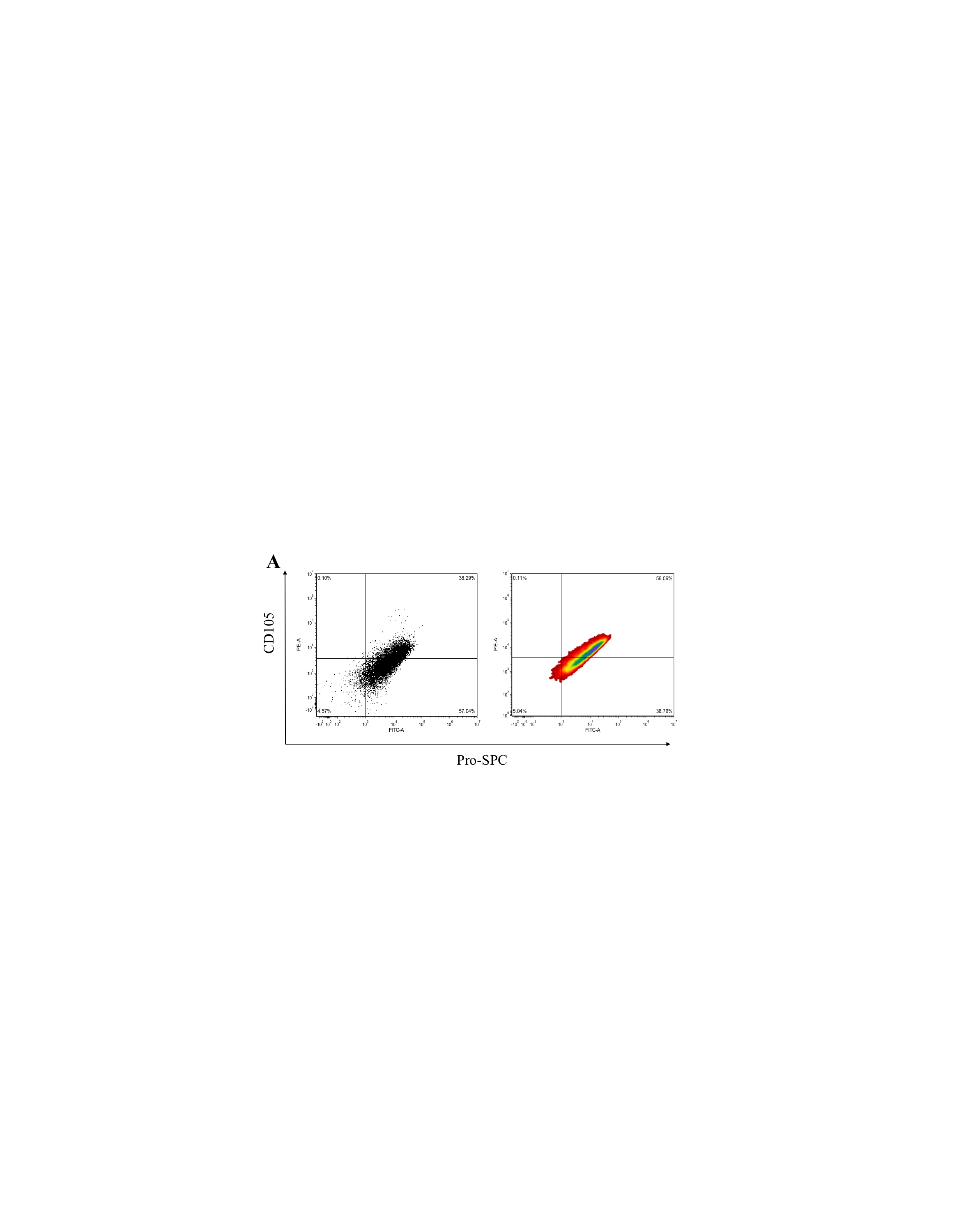

Supplement: Supplementary file 1 — Double stained LSCs shows mixed phenotype of mesenchymal and epithelial markers. [file 12931_2017_611_MOESM1_ESM.tiff]

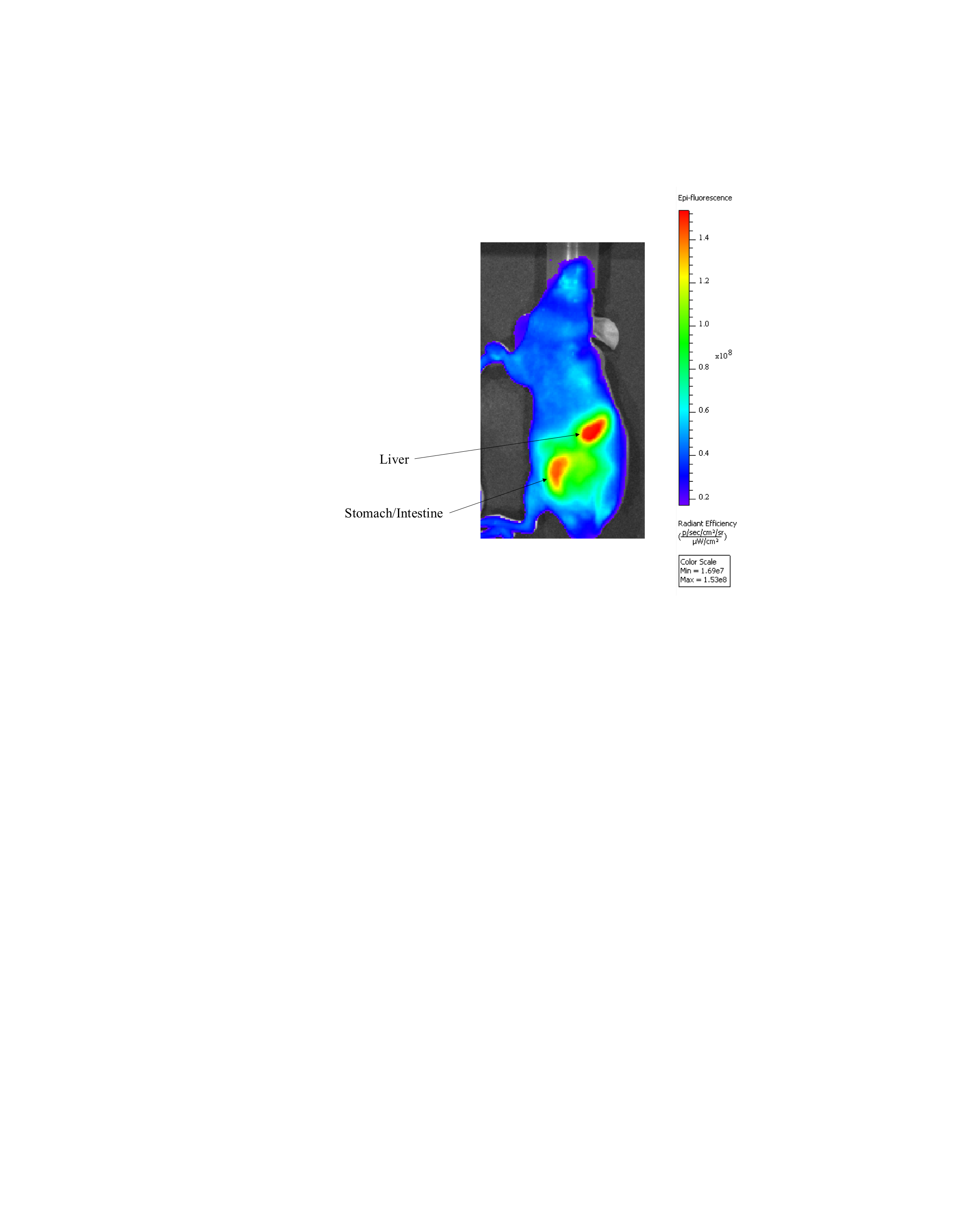

Supplement: Supplementary file 2 — Representative in-vivo imaging of control athymic nude mice showing auto-fluorescence. [file 12931_2017_611_MOESM2_ESM.tiff]

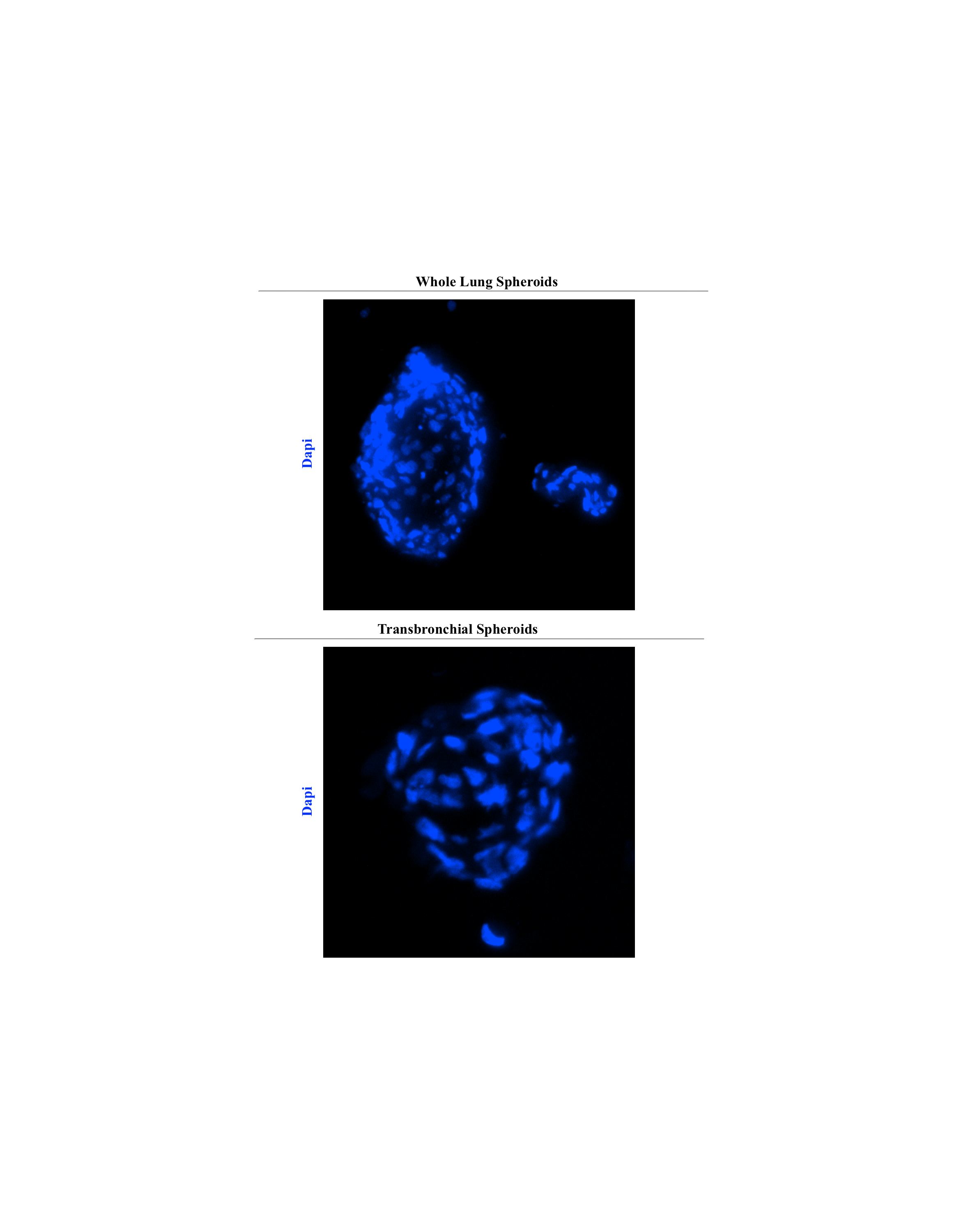

Supplement: Supplementary file 3 — Negative controls of all immunostaining for phenotype analysis of lung spheroids. [file 12931_2017_611_MOESM3_ESM.tiff]

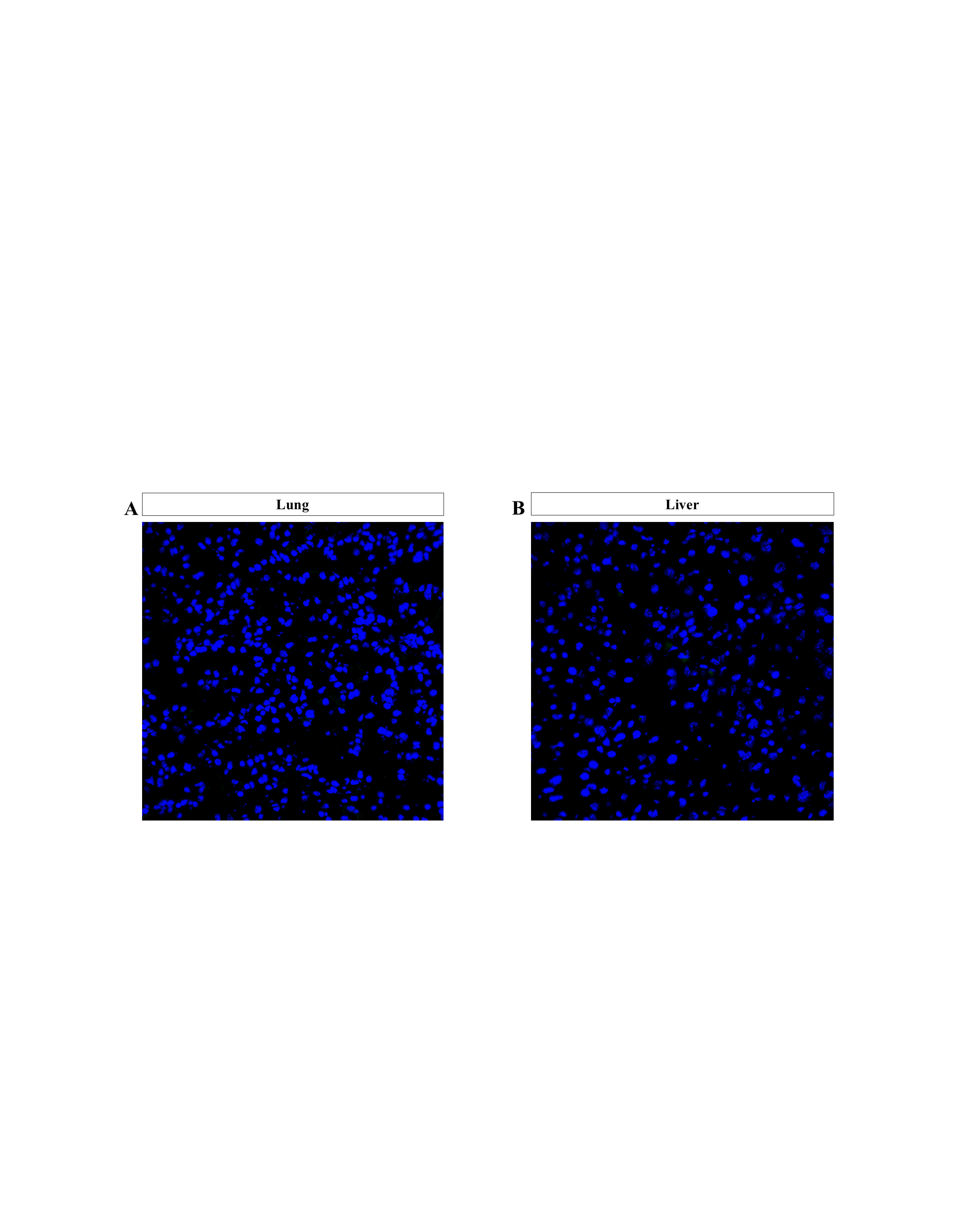

Supplement: Supplementary file 4 — Negative controls of all immunostaining for biodistribution of LSCs in-vivo after intravenous injection. [file 12931_2017_611_MOESM4_ESM.tiff]
